# Deficiency of Tristetraprolin Triggers Hyperthermia through Enhancing Hypothalamic Inflammation

**DOI:** 10.3390/ijms22073328

**Published:** 2021-03-24

**Authors:** Da Yeon Jeong, Nuri Song, Hye Rim Yang, Thai Hien Tu, Byong Seo Park, Hara Kang, Jeong Woo Park, Byung Ju Lee, Sunggu Yang, Jae Geun Kim

**Affiliations:** 1 Division of Life Sciences, College of Life Sciences and Bioengineering, Incheon National University, Incheon 22012, Korea; dayeon@inu.ac.kr (D.Y.J.); sannudeul@naver.com (N.S.); hr.yang0414@inu.ac.kr (H.R.Y.); thaihientu@gmail.com (T.H.T.); bbs0808@naver.com (B.S.P.); harakang@inu.ac.kr (H.K.); 2 Department of Biological Science, University of Ulsan, Ulsan 44610, Korea; jwpark@ulsan.ac.kr (J.W.P.); bjlee@ulsan.ac.kr (B.J.L.); 3 Department of Nano-Bioengineering, Incheon National University, Incheon 22012, Korea

**Keywords:** tristetraprolin, hypothalamus, inflammation, microglia, sickness response, hyperthermia, energy metabolism

## Abstract

Tristetraprolin (TTP), an RNA-binding protein, controls the stability of RNA by capturing AU-rich elements on their target genes. It has recently been identified that TTP serves as an anti-inflammatory protein by guiding the unstable mRNAs of pro-inflammatory proteins in multiple cells. However, it has not yet been investigated whether TTP affects the inflammatory responses in the hypothalamus. Since hypothalamic inflammation is tightly coupled to the disturbance of energy homeostasis, we designed the current study to investigate whether TTP regulates hypothalamic inflammation and thereby affects energy metabolism by utilizing TTP-deficient mice. We observed that deficiency of TTP led to enhanced hypothalamic inflammation via stimulation of a variety of pro-inflammatory genes. In addition, microglial activation occurred in the hypothalamus, which was accompanied by an enhanced inflammatory response. In line with these molecular and cellular observations, we finally confirmed that deficiency of TTP results in elevated core body temperature and energy expenditure. Taken together, our findings unmask novel roles of hypothalamic TTP on energy metabolism, which is linked to inflammatory responses in hypothalamic microglial cells.

## 1. Introduction

The hypothalamus is the central unit that controls multiple body homeostasis [1]. In particular, whole-body energy homeostasis is tightly coupled to the operation of the hypothalamic circuit through the integration of hormonal signals and neuronal inputs [2]. Thus, it is currently well accepted that deterioration of hypothalamic circuit activity causes the development of metabolic diseases such as obesity and diabetes [3]. A growing body of evidence has firmly suggested that hypothalamic inflammation in association with overnutrition could be a primary pathological cellular event in the acceleration of positive energy balance and obesity development [4]. Since microglia, the smallest glial cells, are similar in nature to macrophages in the peripheral immune system and serve as a representative of the brain immune system, multiple lines of evidence have suggested that hypothalamic microglia actively participate in the development and deterioration of hypothalamic inflammation [5,6]. During the past decade, a great deal of attention has been paid to investigating the interrelationship between chronic hypothalamic inflammation and obesity pathogenesis; however, whether an acute hypothalamic inflammation governed by microglial cells is coupled to negative energy balance and sickness behaviors, including anorexia, hyperthermia, and hypoactivity, needs to be clarified [7].

Post-transcriptional regulation of inflammatory genes is also a crucial molecular event in inflammatory responses and is tightly connected to various inflammation-associated diseases. In this regard, RNA-binding proteins (RBPs), which control the stability of RNA by recognizing 3′ untranslated regions (UTRs) of target mRNAs, determine the fate of target mRNAs involved in the pro-inflammatory responses [8,9]. Tristetraporlin (TTP), an RNA-binding protein, governs the stability of the target mRNAs by capturing the AU-rich elements (AREs) in the 3′-UTRs of their target mRNAs [10]. Previous studies have shown that TTP displays anti-inflammatory properties and functions by promoting the degradation of major pro-inflammatory cytokines such as tumor necrosis factor-α (TNF-α), interleukin-1-β (IL-1β), and cyclooxygenase-2 (Cox-2), an enzyme involved in the synthesis of prostaglandins [11,12]. Furthermore, TTP deficient mice display multiple inflammatory diseases accompanied by the overproduction of inflammatory cytokines through a prolonged half-life of target mRNAs involved in pro-inflammatory responses [13,14]. However, it has not yet been identified whether TTP plays an active role in the regulation of inflammatory responses in the hypothalamic microglia and, in turn, controls the development of negative energy balance. Therefore, the current study aimed to evaluate the effect of TTP on the development of inflammatory responses in the hypothalamic microglia and further identified the pathophysiological relevance by determining the metabolic phenotypes involved in the sickness responses triggered by hypothalamic inflammation.

## 2. Results

### 2.1. Hypothalamic TTP Responded to Inflammatory Stimuli

**Figure 1:**
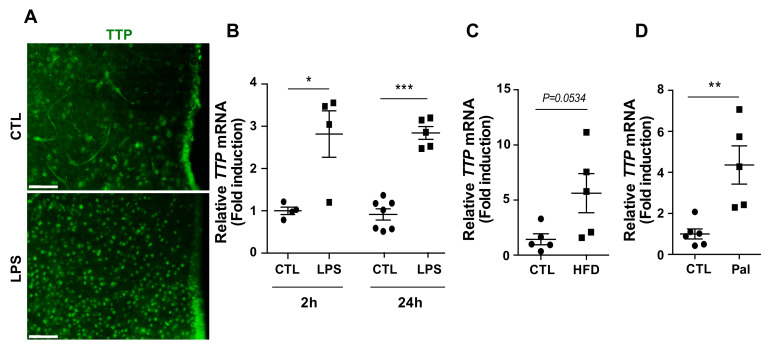
Elevation of Tristetraporlin (TTP) protein and mRNA expression levels by multiple inflammatory stimuli. (**A**) Representative image showing increased signals of TTP protein in the hypothalamus upon lipopolysaccharide (LPS) intraperitoneal (i.p.) injection into C57BL/6 mice as determined by immunohistochemistry. qPCR data show elevated *TTP* mRNA in response to inflammatory stimulants including (**B**) lipopolysaccharide (LPS, treated for 2 h or 24 h), (**C**) HFD (high-fat diet), and (**D**) Palmitic acid (Pal). Data are presented as the mean ± SEM. *n* = 4–6 mice per group for LPS treatment; *n* = 5 mice per group for HFD treatment; *n* = 5–6 mice per group for palmitic acid treatment. * *p* < 0.05, ** *p* < 0.01, and *** *p* < 0.001. Scale bar = 100 μm. SEM; standard error of the mean.

We first confirmed whether hypothalamic TTP responds to inflammatory stimuli. Fluorescence immunohistochemistry (IHC) data showed that immunosignals of TTP were elevated in the hypothalamus of mice that received intraperitoneal (i.p.) injection of lipopolysaccharide (LPS) (Figure 1A), well known as an endotoxin and inflammatory stimulant. Consistent with the histological data, we also observed elevated mRNA levels of *TTP* in response to i.p. injection of LPS (Figure 1B). Since it has been well established that overnutrition triggers hypothalamic inflammation, we next evaluated the mRNA expression of *TTP* in the total hypothalamus of mice fed a high-fat diet (HFD) for 8 weeks and found that mice fed a HFD displayed increased mRNA levels of *TTP* in the hypothalamus compared to that of wild-type mice (Figure 1C). In accordance with these data, we further identified that the mRNA level of *TTP* was increased by i.p. administration of palmitate, a saturated fatty acid (Figure 1D), which leads to an inflammatory response during exposure to a fat-rich diet. Based on these results, we hypothesized that TTP might be functionally linked to the development of hypothalamic inflammation and related pathological outputs.

### 2.2. Deficiency of TTP Led to an Enhanced Inflammatory Response in the Hypothalamus

**Figure 2:**
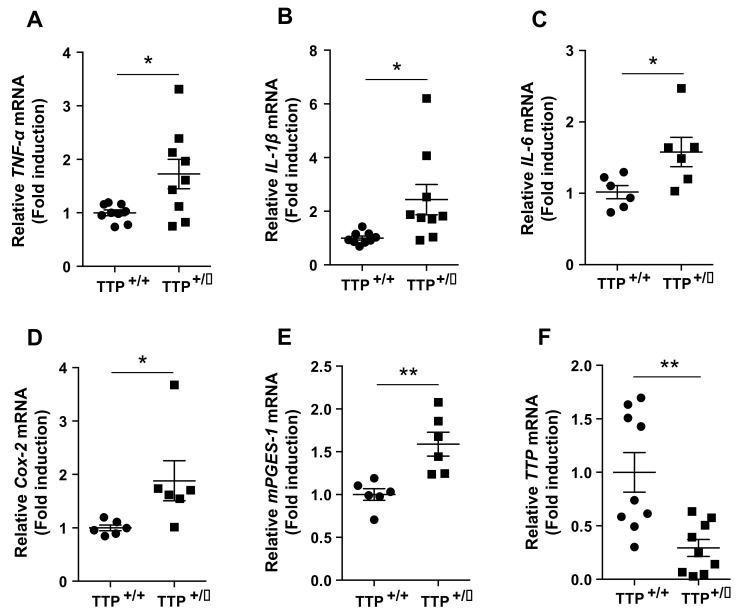
Upregulation of pro-inflammatory genes in the hypothalamus of TTP-deficient mice. Elevated mRNA levels of pro-inflammatory cytokines including (**A**) *TNF-*α, (**B**) *IL-1 β*, (**C**) *IL-6*, and genes involved in the synthesis of prostaglandin E_2_, including (**D**) *Cox-2* and (**E**) *mPGES-1* in the hypothalamus of the TTP-deficient (TTP +/−) mice compared to that of wild type (WT) mice (TTP +/+). (**F**) qPCR data reveal a significant reduction in *TTP* mRNA in the hypothalamus of the TTP-deficient mice compared with that in WT mice. Data are presented as the mean ± SEM. *n* = 9 mice per group for Figure 2A,B,F; *n* = 6 mice per group for Figure 2C–E. * *p* < 0.05 and ** *p* < 0.01. SEM; standard error of the mean.

We next evaluated the mRNA expression of pro-inflammatory genes in the hypothalamus utilizing TTP-deficient mice. qPCR analysis revealed elevated mRNA levels of pro-inflammatory cytokines, including *TNF-α* (Figure 2A), *IL-1β* (Figure 2B), and *interleukin-6 (IL-6)* (Figure 2C), and genes regulating the synthesis of prostaglandins, which also retain proinflammatory properties such as *Cox-2* (Figure 2D) and *microsomal prostaglandin E synthase-1 (mPGES-1)* (Figure 2E). We also confirmed an effective reduction in the *TTP* gene (Figure 2F) in the hypothalamus of TTP-deficient mice compared with wild-type mice. These data suggest that TTP participates in the pathophysiology of hypothalamic inflammation.

### 2.3. Deficiency of TTP Elicited Enhanced Inflammatory Responses in the Hypothalamic Microglia

**Figure 3:**
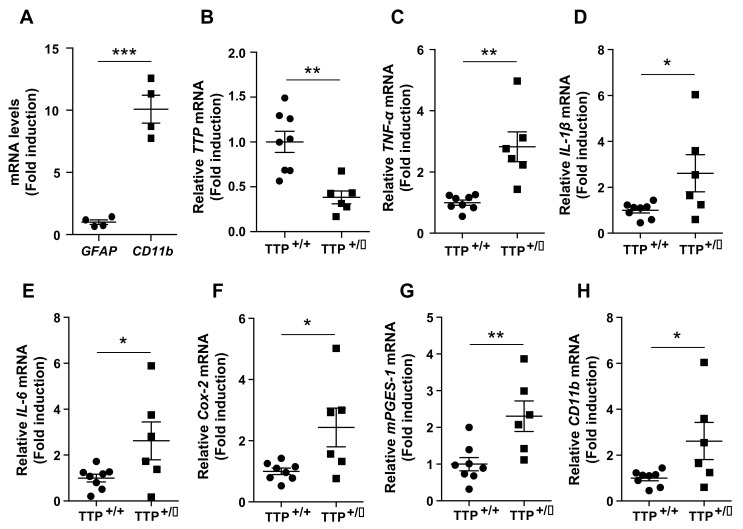
Deficiency of TTP leads to increased expression levels of genes involved in inflammatory responses. Primary microglia were extracted from the hypothalamus of both TTP-deficient and wild-type mice and seeded at 5×10^5^ cells/well to determine mRNA expression. (**A**) Cultured hypothalamic primary microglial cells retain a 10-fold higher level of *CD11b* mRNA compared to that of *Gfap*. (**B**) qPCR data revealed a significant decrease in *TTP* mRNA in the primary microglial cells extracted from the hypothalamus of the TTP-deficient mice compared with that of wild-type mice. qPCR data display elevated mRNA expression of pro-inflammatory cytokines including (**C**) *TNF-*α, (**D**) *IL-1β*, and (**E**) *IL-6*, and genes involved in the synthesis of prostaglandin E_2_, including (**F**) *Cox-*2 and (**G**) *mPGES-1*, and (**H**) *CD11b*, a molecular marker for microglial activation, in the primary microglial cells extracted from the hypothalamus of the TTP-deficient mice compared with that in wild-type control mice. Data are presented as the mean ± SEM. *n* = 4 experiments per group for Figure 3A; *n* = 6–8 experiments per group for Figure 3B–H. All experiments were performed from at least three different preparations of microglial cells. * *p* < 0.05; ** *p* < 0.01; *** *p* < 0.001. SEM; standard error of the mean.

To further confirm whether deficiency of TTP affects the inflammatory tone in the hypothalamic microglia, we evaluated the mRNA expression levels of genes that control inflammation in cultured primary microglia isolated from the hypothalamus of TTP-deficient and wild-type mice. In order to test the purification of primary microglial cells isolated from hypothalamus, we evaluated mRNA expression of genes including *glial fibrillary acidic protein (Gfap)*, a molecular maker for astrocyte and *cluster of differentiation molecule 11b (CD11b)*, a molecular maker for microglia. We confirmed purification of cultured primary microglial cells by identifying an approximately 10-fold higher mRNA level of *CD11b* compared to that of *Gfap* (Figure 3A). In addition, we confirmed a significant reduction in *TTP* mRNA expression in cultured primary microglial cells isolated from the hypothalamus of TTP-deficient mice compared with that of wild-type mice (Figure 3B). In support of identified TTP functions as an anti-inflammatory RBP, we observed that TTP-deficient mice displayed an increase in mRNA levels of pro-inflammatory cytokines such as *TNF-α* (Figure 3C), *IL-1β* (Figure 3D) and *IL-6* (Figure 3E) in cultured hypothalamic microglia when compared with that of wild-type mice. In accordance with mRNA data, we further identified that the release of pro-inflammatory cytokines including *IL-1β* and *IL-6* was increased in the medium of the cultured primary microglial cells extracted from the hypothalamus of TTP-deficient mice when compared with that of wild-type mice (Supplementary Figure S2). Moreover, we found that the mRNA levels of *Cox-2* (Figure 3F) and *mPGES-1* (Figure 3G) were significantly elevated in the cultured hypothalamic microglial cells of TTP-deficient mice compared with that of wild-type mice. We further observed that TTP-deficient mice showed increased mRNA levels of *CD11b*, which can be used as a molecular marker for microglial activation [15,16] (Figure 3H). From these results, we verified that TTP plays an active role in controlling the inflammatory processes in hypothalamic microglia.

### 2.4. Microglial Activation Was Observed in the Hypothalamus of TTP Deficient Mice

**Figure 4:**
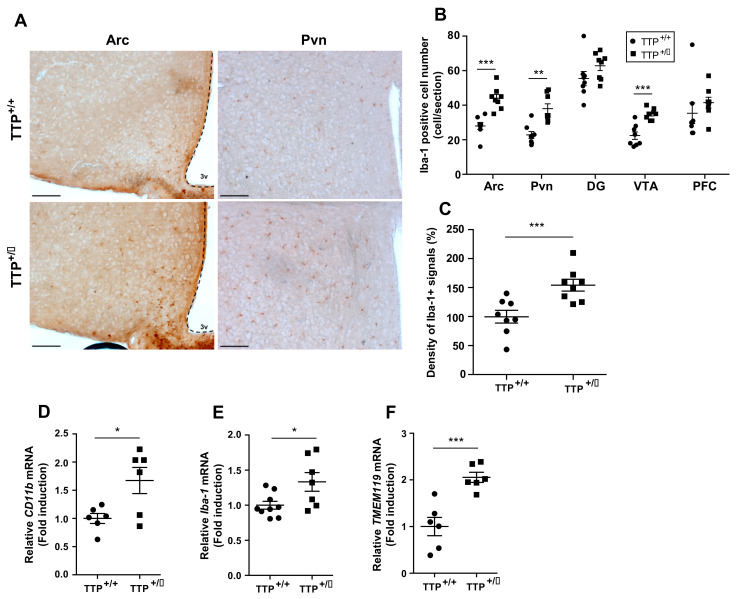
Deficiency of TTP leads to the activation of microglia in the hypothalamus. (**A**) Representative images show immunosignals of Iba-1 protein, a molecular marker for microglial cells, in the hypothalamus as determined by IHC. (**B**) Iba-1 positive cells were significantly elevated in hypothalamic arcuate nucleus (Arc), paraventricular nucleus (Pvn) and ventral tegmental area (VTA), but not significantly altered in the hippocampal dentate gyrus (DG) and prefrontal cortex (PFC) of the TTP-deficient mice compared to that of wild type mice. (**C**) A significant increase in the intensity of Iba-1 immunosignals was observed in the hypothalamic Arc of the TTP-deficient mice compared to that of wild type mice as determined by IHC. qPCR data reveal increased levels of (**D**) *CD11b*, (**E**) *Iba-1*, and (**F**) *TMEM119*, molecular markers for microglia activation, in total hypothalamus of the TTP-deficient mice compared to that of wild type mice. Data are presented as the mean ± SEM. *n* = 8 mice per group for IHC data (Figure 4A–C); *n* = 6–9 mice per group for qPCR data (Figure 4D–F). * *p* < 0.05, ** *p* < 0.01. *** *p* < 0.001. Scale bar = 100 μm. SEM; standard error of the mean.

Different studies have suggested that microglial activation is the hallmark of the development of brain inflammation [17]. In addition, hypothalamic microgliosis accompanied by sustained inflammation is tightly coupled to metabolic disorders such as obesity and dyslipidemia [18,19,20,21]. Thus, we additionally evaluated the distribution of microglia in the hypothalamic regions as determined by IHC experiments utilizing an antibody against ionized calcium-binding adapter molecule 1(Iba-1) protein, which is a molecular marker for activated microglia, where it participates in membrane ruffling and phagocytosis. Since an increased number of microglia occurred when they were activated, we first determined the number of cells retaining Iba-1 immunosignals in the arcuate nucleus (Arc), a center for integrating energy homeostasis by mediating peripheral afferent inputs, and the paraventricular nucleus (Pvn), a center for regulating energy expenditure by operating both sympathetic nerve activity and secretion of endocrine hormones in the hypothalamus (Figure 4A). Consistent with the mRNA data obtained from total hypothalamus and primary microglia, we found an increased number of microglial cells in the hypothalamic Arc and Pvn of TTP-deficient mice compared with that of wild-type mice. Intriguingly, a significant elevation in the number of microglial cells was observed in the ventral tegmental area (VTA) of TTP-deficient mice compared with that of wild type mice (Figure 4B). However, no significant alteration in the number of microglial cells was found in the hippocampal dentate gyrus (DG) and prefrontal cortex (PFC) (Figure 4B). We additionally identified that the intensity of Iba-1 immunosignals was significantly increased in the hypothalamus of the TTP-deficient mice compared with that of the wild-type mice (Figure 4C). These data suggest that TTP plays an active role in inflammation in hypothalamic nuclei and VTA rather than in other regions of the brain. In accordance with the histology data, we further observed that TTP-deficient mice showed elevated mRNA expression levels of *CD11b*, *Iba-1*, and *transmembrane protein 119(TMEM119)*, which are molecules that actively participate in the activation of microglia in the hypothalamus (Figure 4D–F). Taken together, a reduction in TTP leads to enhanced microglial activation accompanied by inflammatory responses in the hypothalamic nuclei and VTA.

### 2.5. Deficiency of TTP Led to Altered Neuronal Activity in the Hypothalamic Arc

**Figure 5:**
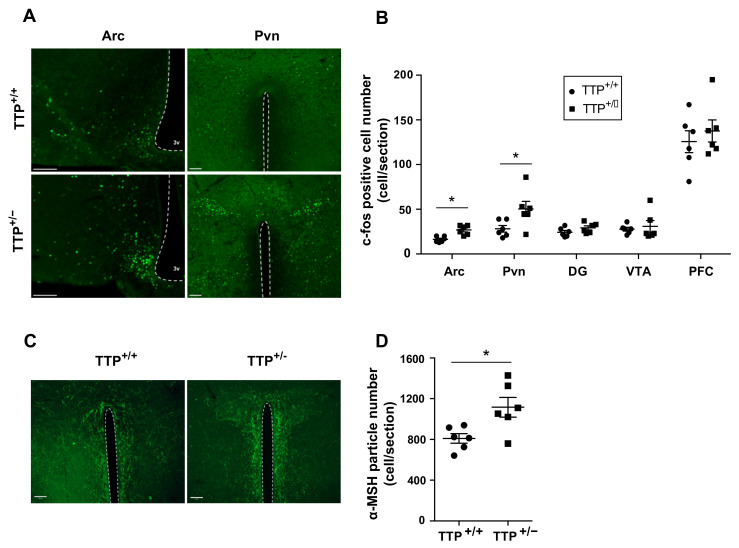
Deficiency of TTP leads to enhanced immunosignals of c-fos and alpha-melanocyte-stimulating hormone (MSH) fibers in the hypothalamic Arc and Pvn. (**A**) Representative images reveal immunosignals of c-fos, a marker for neuronal activation, in the hypothalamic Arc and Pvn as determined by IHC. (**B**) A higher number of c-fos positive cells in the hypothalamic Arc and Pvn of the TTP-deficient mice compared to that of wild type mice. No significant alteration of c-fos positive cells was found in the DG, VTA and PFC of the TTP-deficient mice compared to that of wild type mice. (**C**) Representative images obtained by IHC show increased immunosignals of α-MSH fibers in the hypothalamic Pvn. (**D**) The number of α-MSH fiber signals was elevated in hypothalamic Pvn of the TTP-deficient mice compared to that in wild type mice. Data are presented as the mean ± SEM. *n* = 6 mice per group. * *p* < 0.05. Scale bar = 100 μm. SEM; standard error of the mean.

Hypothalamic microgliosis linked to elevated circulating levels of fatty acid affects the operation of hypothalamic neurons controlling appetite and energy expenditure such as agouti-related peptide (Agrp) and proopiomelanocortin (Pomc)-positive neurons [22,23,24]. Therefore, we evaluated the neuronal activity in the hypothalamic Arc, where Agrp and Pomc neurons are predominantly present, and hypothalamic Pvn, one of the major target nuclei of melanocortin signals derived from Pomc neurons, to determine whether the melanocortin pathway is involved in the induction of microglial activation caused by TTP downregulation. Intriguingly, we observed that TTP-deficient mice showed a significant increase in immunosignals of c-fos, an immediate early gene that is regarded as a molecular marker for enhanced neuronal excitability, in both hypothalamic Arc and Pvn (Figure 5A,B). However, no significant alteration of c-fos signals was observed in the hippocampal DG, VTA and prefrontal cortex (PFC). Since alpha-melanocyte-stimulating hormone (alpha-MSH) originates from the Pomc gene by proteolytic cleavage and targets Pvn, where efferent POMC neurons trigger satiety signals and enhance energy expenditure, we further evaluated the signals of alpha-MSH fibers in Pvn of both wild type and TTP-deficient mice. We found that TTP-deficient mice displayed an increased number of alpha-MSH fibers in Pvn compared to wild-type mice (Figure 5C,D). These observations suggest that TTP-controlled hypothalamic inflammation is coupled to the operation of the hypothalamic melanocortin system.

### 2.6. Hyperthermia Occurred in TTP-Deficient Mice

**Figure 6:**
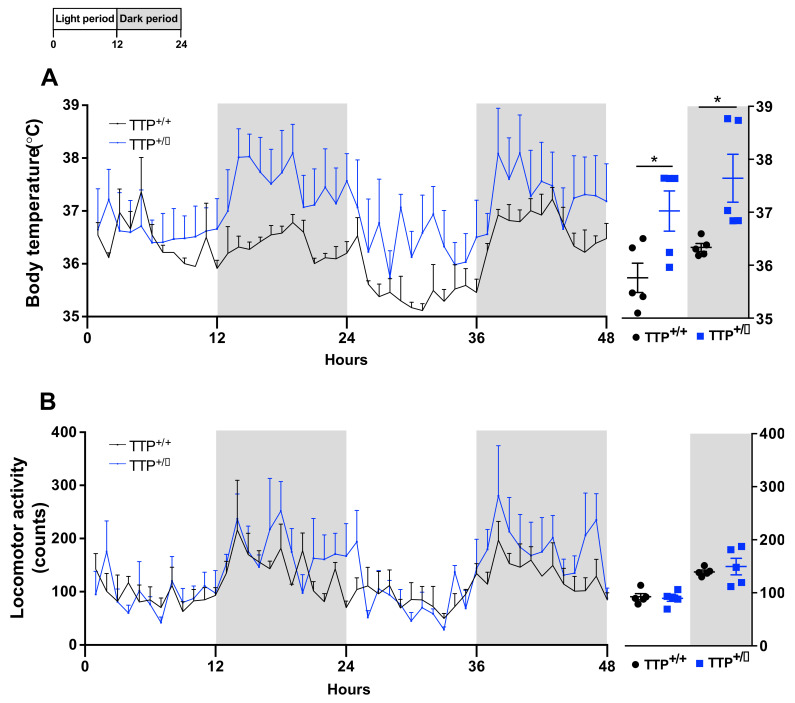
TTP-deficient mice display signs of hyperthermia. Core body temperature and physical activity were determined using telemetry probes. (**A**) An elevation of core body temperature was observed in the TTP-deficient mice compared to that in wild-type mice. (**B**) Locomotor activity was not altered in the TTP-deficient mice compared to that in wild-type control mice. Data are presented as the mean ± SEM. *n* = 5 mice per group. * *p* < 0.05. SEM; standard error of the mean.

In order to identify the pathophysiological relevance of the cellular and molecular observations obtained from TTP-deficient mice, both TTP deficient and wild-type mice were subjected to evaluation of the core-body temperature (Figure 6A) and physical activity (Figure 6B), which are the behavioral outputs in association with hypothalamic inflammation and altered melanocortin tone in the hypothalamus. In accordance with the enhanced hypothalamic inflammatory response, an elevated core-body temperature was observed in TTP-deficient mice during both light and dark periods. However, no alteration in locomotive activity was observed, which is also one of the behavioral phenotypes associated with hypothalamic inflammation. Taken together, these observations suggest that TTP has a functional contribution to the hypothalamic control of thermogenesis during the development of inflammatory processes.

### 2.7. TTP-Deficient Mice Displayed Increased Energy Expenditure

**Figure 7:**
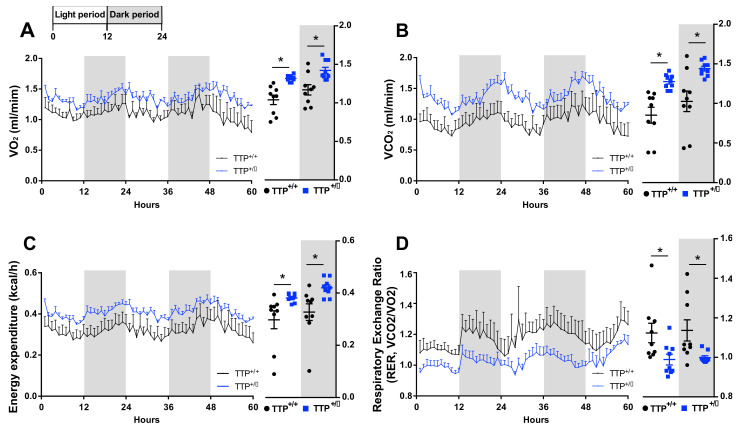
TTP-deficient mice display increased energy expenditure and lipid utilization. Metabolic phenotypes that reflect energy expenditure were determined using an indirect calorimetry instrument. (**A**) Increased oxygen consumption (VO_2_), (**B**) carbon dioxide generation (VCO_2_), and (**C**) energy expenditure were observed in TTP-deficient mice compared to that in wild-type mice. (**D**) Respiratory exchange ratio (RER) was decreased in the TTP-deficient mice compared to that in wild-type mice. Data are presented as the mean ± SEM. *n* = 9 mice per group. * *p* < 0.05. SEM; standard error of the mean.

We further investigated the pattern of energy expenditure in both wild type and TTP-deficient mice using an indirect calorimetry instrument. TTP-deficient mice showed an increase in energy expenditure accompanied by higher oxygen consumption (VO_2_) and carbon dioxide production (VCO_2_) (Figure 7A–C), which is consistent with previous reports showing increased energy expenditure during hypothalamic inflammation [25]. Respiratory exchange ratio (RER), the ratio between the amount of CO_2_ emission and O_2_ consumption, can be used to determine the proportion of carbohydrates and fats utilized. TTP-deficient mice showed a reduction in the RER value, indicating enhanced lipid utilization (Figure 7D). This result implies that enhanced thermogenic effects observed in TTP-deficient mice might be linked to increased lipid utilization. Overall, our findings suggest that the phenotypes of energy expenditure seen in TTP-deficient mice can be explained, at least in part, via the molecular and cellular observations showing enhanced hypothalamic inflammation. 

### 2.8. Deficiency of TTP Led to Hyperphagia without Body Weight Change

**Figure 8:**
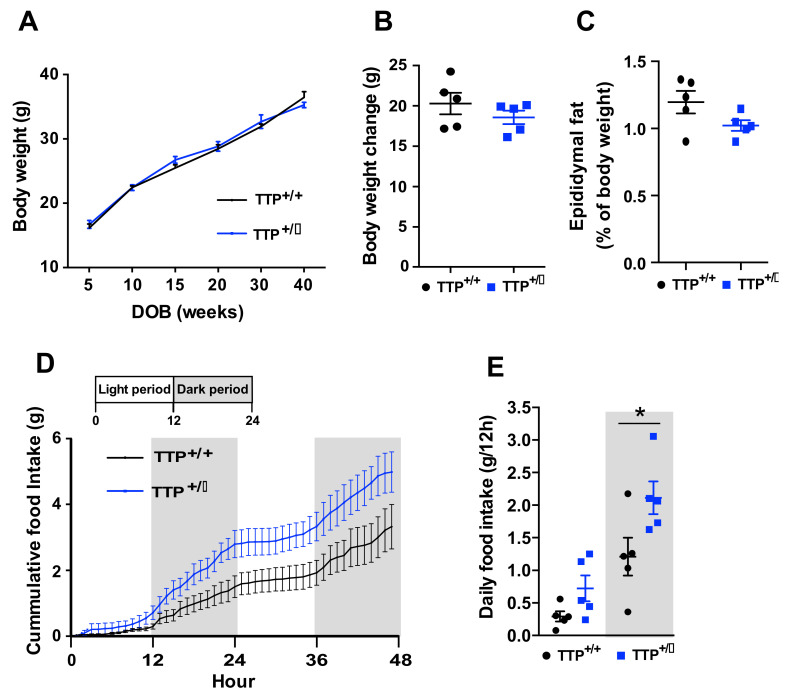
Deficiency of TTP results in an increase in food intake and no alteration of body weight. Body weight changes were measured in both the TTP-deficient mice and wild-type mice under standard diet treatment. (**A**) No significant difference in body weight and (**C**) adiposity (ratio of epididymal fat weight per body weight) was found between TTP-deficient mice and wild type mice during the observation periods (from 5-week-old to 40-week-old). (**B**) Cumulative body weight gain for 36 weeks was not significantly altered between the TTP-deficient mice and wild-type mice. (**D**) Cumulative food intake for 48 h tended to be higher in the TTP-deficient mice (10-week-old) than in wild-type mice. (**E**) Average food intake for 12 h was enhanced in the TTP-deficient mice compared to that in wild-type mice. Data are presented as the mean ± SEM. *n* = 5 mice per group. * *p* < 0.05. SEM; standard error of the mean.

To further identify whether enhanced thermogenesis and energy expenditure were linked to changes in body weight and food intake, we compared the changes in the body weight and food intake between TTP-deficient and wild-type mice. Although anorexia and body weight loss can be observed during the development of negative energy balance, TTP-deficient mice paradoxically displayed no significant difference of body weight (Figure 8A,B) and adiposity (Figure 8C), and a tendency toward increased food intake (Figure 8D,E). These data imply that TTP may participate in the regulation of appetite regardless of inflammation-induced anorexia or that TTP-deficient mice displayed hyperphagic behavior to compensate for the loss of adiposity induced by enhanced thermogenesis and lipid utilization.

## 3. Discussion

The present study highlighted the previously underappreciated function of TTP in the central nervous system. We demonstrated that genetic ablation of TTP led to the development of hypothalamic inflammation coupled to microglial activation and pathological outputs, including hyperthermia and increased energy expenditure. A great deal of attention has been paid to investigating intracellular signaling molecules that mediate extracellular inflammatory stimuli, including external environment-derived pathogens and internal substances such as hormones, cytokines, and nutrients. Among the multiple molecular components, transcription factors act as a final switch to control the expression of pro-inflammatory genes [26]. For instance, the transcription factor nuclear kappa B (NF-*κ*B) plays a major role in innate immunity and inflammatory responses, and is regarded as a major link between inflammation and related diseases [27]. Recently, intensive research has been conducted to investigate the post-transcriptional regulators involved in pro-inflammatory and anti-inflammatory processes. In support of this notion, it has been established that RNA-binding proteins (RBPs) also participate in post-transcriptional regulation of genes associated with innate immunity by modulating the stability of their target RNAs [28]. 

The hypothalamus is the central unit that governs a variety of body homeostasis [1]. In particular, the circuit activity in hypothalamic nuclei operates the peripheral metabolic organs such as adipose tissues and thus maintains the whole-body energy homeostasis [29]. Furthermore, hypothalamic inflammation has been regarded as a critical pathological event in the disruption of energy balance, causing obesity pathogenesis [30]. In this context, it is not surprising that evidence has emerged linking the development of hypothalamic inflammation and the functions of RBPs in the post-transcriptional regulation of inflammatory genes. In order to confirm our working hypothesis, we mainly utilized the global TTP-deficient mice due to severe symptoms involved in inflammatory responses seen in global TTP knock-out mice [13]. Consistent with the previous findings showing enhanced peripheral innate immunity seen in TTP knock-out mice, TTP-deficient mice showed enhanced hypothalamic inflammation as well as elevated circulating levels of proinflammatory cytokines (Supplementary Figure S1). Although glial cells are the most abundant cells and are considered to be primary orchestrators for neuronal functions beyond their contributions to support neuronal homeostasis [31,32,33], the effects of non-neuronal cells have been relatively relegated to a less prominent role in the control of energy homeostasis supported by hypothalamic neurocircuitry. Among the glial cells, microglia is well identified as an active participant in the development and deterioration of hypothalamic inflammation [19]. We also verified that microglial activation is a major cellular event to interpret the development of hypothalamic inflammation in TTP down-regulation. Notably, it has been determined that the inflammatory processes in microglia give rise to the perturbation of hypothalamic neurocircuitry and thus, cause abnormalities in whole-body energy metabolism [17]. In addition, it has been proposed that functional interactions between hypothalamic neurons and microglia determine metabolic phenotypes, including feeding and energy consumption. In support of this evidence, the present study also confirmed that TTP-deficient mice showed altered neuronal excitability in the hypothalamic nuclei where presynaptic and postsynaptic melanocortin components exist. Previous studies have suggested that acute hypothalamic inflammation, irrespective of overnutrition and/or elevated nutritional components, is implicated in the occurrence of a negative energy balance accompanied by sickness behaviors, including anorexia, hyperthermia, and hypoactivity [34]. In addition, our previous study has also proposed that Toll-like receptor 2 signaling triggers sickness behaviors by modulating the microglia–neuronal axis in the hypothalamus [7]. In accordance with this evidence and our molecular and cellular observations obtained from TTP -deficient mice, our study provides experimental clues that could propose the pathophysiological roles of hypothalamic TTP with the determination of multiple behavioral phenotypes such as hyperthermia and increased energy expenditure observed during hypothalamic inflammation. Intriguingly, TTP-deficient mice displayed increased lipid utilization as assessed by a reduction in the RER value as well as increased energy expenditure, which are the general behavioral outputs reflecting the status of negative energy balance. However, TTP-deficient mice did not show any change in body weight, even though elevated core-body temperature and energy expenditure were detected. Surprisingly, the TTP-deficient mice displayed a slightly increased food intake. This paradoxical hyperphagic response might be due to compensatory feeding behavior to maintain energy balance. Presumably, the existence of direct regulatory roles of TTP in hunger-promoting neurons might be another possible explanation for unchanged body weight. Therefore, further studies are needed to clarify the cell-specific roles of hypothalamic TTP in the control of whole-body energy metabolism. 

Collectively, the current study identified the active roles of TTP in hypothalamic inflammation in association with microglial activation and in the promotion of hyperthermia and increased energy expenditure. These findings provide a novel insight into the molecular mechanism linked to the development of hypothalamic inflammation to identify strategies that can be utilized to treat patients with severe sickness responses.

## 4. Materials and Methods

### 4.1. Animals

Tristetraprolin deficient (TTP +/−) mice (generated by Dr. Perry J. Blackshear, National Institute of Environmental Health Sciences, Durham, NC, USA) were fed standard chow (DBL, Chungcheongbuk-do, Korea) *ad libitum*, unless otherwise stated and maintained in a temperature and humidity-controlled room with a 12 h–12 h light–dark cycle, with lights on from 7:00 a.m. to 7:00 p.m. Lipopolysaccharide (LPS, 100 μg/kg, Sigma-Aldrich, St. Louis, MO, USA) was administered to 8-week-old C57BL/6 mice by an intraperitoneal (i.p.) injection. Palmitic acid (20 μM, Sigma-Aldrich) was also administered to 8-week-old C57BL/6 mice with i.p. injection daily for 3 days. C57BL/6 mice were fed with a high-fat diet (HFD, 60% of fat calories, Research Diets Inc., New Brunswick, NJ, USA) for 8 weeks and subjected to analysis of *TTP* mRNA expression in the hypothalamus. All animal care and experimental procedures were performed in accordance with a protocol approved by the Institutional Animal Care and Use Committee (IACUC) at the Incheon National University (Permission number: INU-2016-001).

### 4.2. Primary Microglia Culture

Microglial cultures were prepared as described previously [35]. Briefly, following decapitation of five to seven C57BL/6 mice (five-day-old), the diencephalon was removed under sterile conditions and then triturated in Dulbecco’s modified Eagle′s medium (DMEM) F-12 containing 1% penicillin–streptomycin using a pair of corneal scissors. The cell suspension was filtered through a 100-μm sterile cell strainer to remove debris and fibrous layers. The suspension was centrifuged, and the pellet was resuspended in DMEM F-12 containing 10% fetal bovine serum (FBS) and 1% penicillin–streptomycin. The cells were then grown in this culture medium in 75-cm^3^ culture flasks at 37 °C and 5% CO_2_. When the cells reached confluence (at approximately nine days), microglia were separated from the adhered astrocytes by shaking the culture at approximately 250 rpm for 2 h. The cells were then seeded onto 6-well tissue culture plates, previously coated with poly d-lysine hydrobromide (50 μg/mL), after which they were distributed at 5 × 10^5^ cells/well and incubated at 37 °C with 5% CO_2_. 

### 4.3. Quantitative Real-Time PCR

Total RNA was extracted from the hypothalami according to the Tri-Reagent (Invitrogen, Carlsbad, CA, USA) protocol, and cDNA was synthesized from total RNA using a high-capacity cDNA reverse transcription kit (Intron Biotechnology, Seoul, Korea). The mRNA expression levels were measured using a Bio-Rad CFX 96 Real-Time Detection System (Bio-Rad Laboratories, Hercules, CA, USA) with the SYBR Green Real-time PCR Master Mix Kit (TaKaRa Bio Inc., Foster, CA, USA). The results were analyzed using the CFX Manager software and normalized to the levels of *β-actin*, a housekeeping gene. The primers used were: *IL-1β*, F-AGGGCTGCTTCCAAACCTTTGAC and R-ATACTGCCTGCCTGAAGCTCTTGT; *IL-6*, F-CCACTTCACAAGTCGGAGGCTTA and R-GCAAGTGCATCATCGTTGTTCATAC; *TNF-α*, F-TGGGACAGTGACCTGGACTGT and R-TTCGGAAAGCCCATTTGAGT; *cox-2*, F-TGCTGTACAAGCAGTGGCAA and R- CTCGGGCTCTGATGTAGGTC; *mPGES-1*, F-CTGCTGGTCATCAAGATGTACG and R-TGCCAGATTTTCTCCATGTCG; *TTP*, F-GGATCTCTCTGCCATCTACGA and R-CAGTCAGGCGAGAGGTGAC; *TMEM119*, F-CCTTCACCCAGAGCTGGTTC and R-GGCTACATCCTCCAGGAAGG; *Iba-1*, F-AGCTTTTGGACTGCTGAAGG and R-TTTGGACGGCAGATCCTCATC; *CD11b*, F-CCACTCATTGTGGGCAGCTC and R-GGGCAGCTTCATTCATCATGTC; *GFAP*, F-TCAATGACCGCTTTGCTAGC and R-ACTCGT GCAGCCTTACACAG; *β-actin*, F-TGGAATCCTGTGGCATCCATGAAAC and R-TAAAACGCAGCTCAGTAACAGTAACAGTCCG.

### 4.4. Immunohistochemistry

Mice were anesthetized and perfused transcardially with 0.9 % saline (*w*/*v*), followed by fixation with 4 % paraformaldehyde in phosphate buffer (PB, 0.1 M, pH 7.4). Fixative brains were harvested and post-fixed overnight with 4 % paraformaldehyde in PB. The coronal section (thickness, 50 μm) was prepared using a vibratome (5100 mz Campden Instruments; Leicestershire, UK). After several washes with PB buffer, the sections were preincubated with 0.3 % Triton X-100 (Sigma-Aldrich) for 30 min at room temperature. The sections were then incubated with primary antibodies against rabbit TTP (1:500, abcam, Cambridge, UK) [36], rabbit Iba-1 (1:1000 dilution, Wako, Osaka, Japan) [24], rabbit c-fos (1:1000 dilution, Santa Cruz Biotechnology, Dallas, TX, USA) [37], and sheep alpha-MSH (1:1000 dilution, Millipore, MA, USA) [24] overnight at room temperature. Immunofluorescence was performed with a secondary antibody (Alexa Fluor 488-labeled anti-rabbit antibody; 1:1000 dilution; Invitrogen Life Technologies, Carlsbad, CA, USA) for 2 h at room temperature. To perform diaminobenzidine (DAB)-based IHC, sections were incubated with a biotinylated anti-rabbit secondary antibody (Vector Laboratories, Burlingame, CA, USA) for 2 h. After treatment with the avidin-biotin complexes (ABC) reagent (Vector Laboratories) for 2 h, Iba-1 proteins were specifically stained through the DAB substrate (Vector Laboratories). The sections were then placed on a glass slide, mounted with a mounting solution (Dako North America Inc., Carpinteria, CA, USA), and covered with a coverslip to prevent movement and drying of the sample. Images were acquired by fluorescence microscopy (Axioplan2 Imaging; Carl Zeiss Microimaging Inc., Oberkochen, Baden-Württemberg, Germany). For IHC analyses, sections were anatomically matched with the mouse brain atlas. Both sides of the bilateral brain regions were analyzed for two brain sections per mouse. The numbers of Iba-1-positive microglial cells and c-fos-positive cells were counted using ImageJ 1.47 v software (National Institutes of Health, Bethesda, MD, USA; https://imagej.nih.gov/ij/; accessed on 20 March 2021) by an unbiased observer. The intensity of Iba-1 immunosignals detected by DAB-based IHC was measured in a region of interest from a captured image using ImageJ 1.47 v software.

### 4.5. Measurement of Body Temperature and Locomotor Activity

Body temperature and locomotor activity were measured using biotelemetry transmitters (Mini-Mitter, Bend, OR, USA). Mice (9-week-old) were anesthetized with tribromoethanol and the telemetry transmitter was implanted in the peritoneal cavity 1 week before the experiment. After implantation, the layer of the abdominal wall was sutured, and the skin incision was closed with staples. The output (frequency in Hz) was monitored by a receiver (model RA 1000; Mini-Mitter) placed under each cage. A data acquisition system (Vital View, Mini-Mitter) was used for automatic control of data collection and analysis. Body temperature was recorded at 10-min intervals. Changes in locomotor activity were detected as changes in the position of the implanted transmitter over the receiver board, which resulted in a change in the signal strength and was recorded as a pulse of activity. Activity pulses were counted every 10 min, and average values were calculated after 12 h.

### 4.6. Measurement of VO_2_, VCO_2_, Energy Expenditure and Food Intake

A standard 12 h light/dark cycle was maintained throughout the indirect calorimetry studies. Mice (10-week-old) were acclimated to the cages for 48 h before data collection. Energy expenditure and food intake were measured using a computer controlled indirect calorimetry system (Promethion, Sable Systems, North Las Vegas, NV, USA). Food and water were provided ad libitum, and data were collected for three days after acclimation. Oxygen consumption (VO_2_) and carbon dioxide production (VCO_2_) were measured for each mouse at 10-min intervals. Incurrent air reference values were determined after the analysis of the mice in all cages. The respiratory quotient (RQ) was calculated as the ratio of CO_2_ production to O_2_ consumption. Data acquisition and instrument control were performed using MetaScreen v. 1.6.2 software, and the obtained raw data were processed using ExpeData v. 1.4.3 (Sable Systems) with an analysis script detailing all aspects of data transformation.

### 4.7. Measurement of Cytokine Levels

The cytokine levels in supernatants harvested from cultured primary microglial cells and sera collected from mice were measured using an enzyme-linked immunosorbent assays (ELISA). The assays were conducted using mouse IL-1β and IL-6 DuoSet (R&D Systems, Minneapolis, MN, USA) according to the manufacturer’s instructions.

### 4.8. Statistical Analysis

In the images of the stained tissue, the signals of the target protein were uniformly analyzed using the Image J program. All statistical analyses were performed using GraphPad Prism 6.0 software (GraphPad, San Diego, CA, USA), and differences between the two groups were indicated by performing a two-tailed Student’s t-test. All graphs represent the mean ± SEM.

## Data Availability

All data reported in the manuscript and in the Supplementary materials.

## Supplementary Materials

The following are available online at https://www.mdpi.com/1422-0067/22/7/3328/s1, Figure S1: Elevated circulating levels of pro-inflammatory cytokines were observed in sera of TTP-deficient mice, Figure S2: Deficiency of TTP leads to increased release of pro-inflammatory cytokines in cultured primary microglial cells.
