# Supplementary material for: Deficiency of Tristetraprolin Triggers Hyperthermia through Enhancing Hypothalamic Inflammation
Source: Int J Mol Sci. 2021 Mar 24;22(7):3328. doi: 10.3390/ijms22073328 (PMC8037390; doi:10.3390/ijms22073328)
Supplement: Supplementary file 1 [file ijms-22-03328-s001.pdf]

International Journal of
Molecular Sciences

MDPI

Supplementary Figure 1

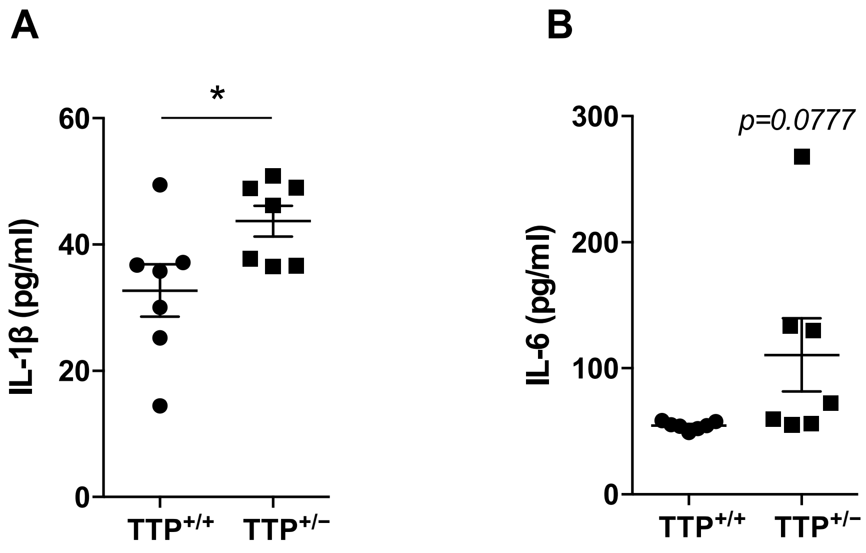

Supplementary Figure1. Elevated circulating levels of pro-inflammatory cytokines were observed in sera of TTP-deficient mice. Circulating levels of IL-1β and IL-6 were measured with sera obtained from both TTP-deficient and wild-type mice by using IL-1β and IL-6 ELISA kits. TTP-deficient mice displayed elevated serum levels of (A) IL-1β and (B) IL-6 compared with wild-type mice. Data are presented as mean ± SEM. *n*= 7 per group. *p<0.05.

Supplementary Figure 2

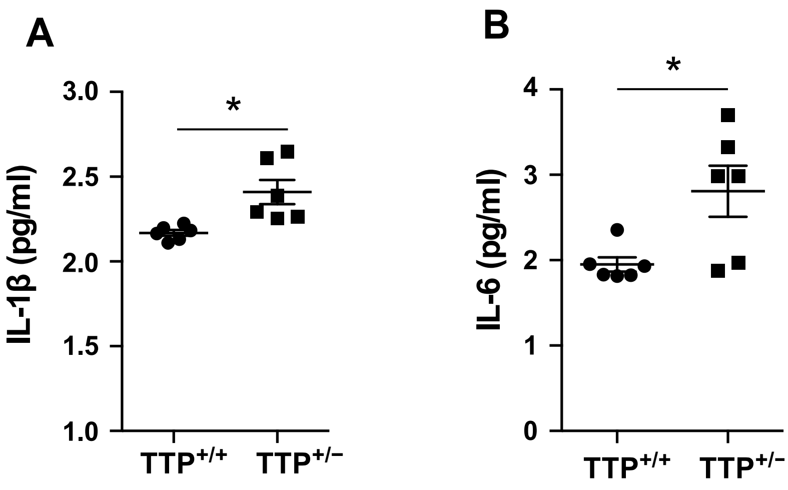

Supplementary Figure 2. Deficiency of TTP leads to increased release of pro-inflammatory cytokines in cultured primary microglial cells.

Primary microglial cells were extracted from hypothalamus of both TTP-deficient and wild-type mice, and seeded at 5×10^5 cells/well to measure medium contents of pro-inflammatory cytokines by using IL-1β and IL-6 ELISA kits. Increased medium levels of (A) IL-1β and (B) IL-6 were observed in cultured primary microglial cells extracted from hypothalamus of TTP-deficient mice compared with that in cultured primary microglial cells extracted from hypothalamus of wild-type mice. Data are presented as mean ± SEM. n=6 per group. *$p<0.05$.
